# Comparison of 4- and 4 plus-courses S-1 administration as adjuvant chemotherapy for pancreatic ductal adenocarcinoma

**DOI:** 10.1186/s12885-021-08380-9

**Published:** 2021-05-26

**Authors:** Bo Li, Shuo Shen, Siting You, Guoxiao Zhang, Suizhi Gao, Xiaohan Shi, Huan Wang, Xiaoyi Yin, Xiongfei Xu, Shiwei Guo, Gang Jin

**Affiliations:** 1 Department of Hepatobiliary Pancreatic Surgery, Changhai Hospital Affiliated to Navy Medical University (Second Military Medical University), 168 Changhai Road, Shanghai, 200433 China; 2 Department of General Surgery, Beidaihe Rehabilitation and Recuperation Center of Joint Logistics Support Force, 4 Xihaitan Road, Qinhuangdao, 066100 China; 3 Central laboratory, Changhai Hospital Affiliated to Navy Medical University (Second Military Medical University), 168 Changhai Road, Shanghai, 200433 China

**Keywords:** Pancreatic ductal adenocarcinoma, Prognosis, S-1, Adjuvant chemotherapy

## Abstract

**Purpose:**

The study aimed to investigate the potential benefit of more than 4 courses of S1 adjuvant chemotherapy for patients with pancreatic ductal adenocarcinoma (PDAC) after surgery.

**Method:**

Data were retrospectively collected from consecutive patients who underwent S-1 adjuvant chemotherapy following curative pancreatectomy between January 2016 and December 2018.

Four-courses and > 4 courses cohorts were compared for overall survival (OS) as a primary outcome, and relapse-free survival (RFS) and adverse event incidence as secondary outcomes.

**Results:**

Four-courses and > 4 courses cohorts comprised 99 patients and 64 ones, respectively. TNM stage (stage II vs. I: HR, 2.125; 95% CI, 1.164–4.213; *P* = 0.015), duration of S-1 administration (4 vs. > 4 courses: HR, 3.113; 95% CI, 1.531–6.327; *P* = 0.002) and tumor grade (G3 vs. G1/2: HR, 3.887; 95% CI, 1.922–7.861; *P* < 0.001) were independent prognostic factors. Under the condition of patients’ survival time beyond 8 months, the OS of patients in > 4 courses cohort was significantly prolonged compared with that of 4 courses cohort (4 vs. > 4 courses: HR, 2.284; 95% CI, 1.197–4.358; *P* = 0.012), especially for patients in TNM stageII (4 vs. > 4 courses: HR, 2.906; 95% CI, 1.275–6.623; *P* = 0.011).RFS and adverse events incidence did not signifcantly difer between both cohorts.

**Conclusion:**

Prolonged duration of S-1 intake is beneficial to prognosis of patients with PDAC resection.

**Supplementary Information:**

The online version contains supplementary material available at 10.1186/s12885-021-08380-9.

## Introduction

Pancreatic ductal adenocarcinoma (PDAC) is the fourth leading cause of cancer-related death worldwide, with a 5-year survival rate of approximately 9% [1]. Surgical resection, followed by adjuvant chemotherapy [2], remains the only potentially curative treatment [3], but only a minority of patients is diagnosed with locally resectable, non-metastatic disease [4]. And 5-year survival rate of those who with local disease could undergo surgery is also extremely lower than that of the other solid tumors [1]. Currently, even after margin-negative resections and favorable pathological staging, the 5-year survival is still about 20% [5]. Due to the tendency of systemic recurrence [6], incorporation of chemotherapy and neoadjuvant therapy [7] has become an intensive treatment, and multiple randomised controlled trials (RCTs) have identified the survival benefit of systemic chemotherapy [5]. The Japan Adjuvant Study Group of Pancreatic Cancer (JASPAC) 01 trial reported that superior overall survival (OS) and relapse-free survival (RFS) in patients who received S-1, an oral 5-fluorouracil prodrug that consists of tegafur (a prodrug of 5-FU), gimeracil (a potent dihydropyrimidine dehydrogenase inhibitor) and oteracil (an inhibitor of phosphorylation of 5-FU in the gastrointestinal tract) in a 1:0.4:1 M concentration ratio, compared with those who received gemcitabine (hazard ratio, 0.57 and 0.60; *P* < 0.001 and *P* < 0.001, respectively) [7]. In addition, previous studies have shown that S-1 or modifified FOLFIRINOX (mFFX) was better to Gemcitabine/Capecitabine in adjuvant treatment of PDAC, which improved the prognosis after surgical resection [5]. It should be considered as a reasonable standard scheme in the adjuvant setting and as control arm for future adjuvant clinical trials [5]. Whilst there were no signifificant difference between S-1 and mFFX for OS, S-1 had signifificantly longer RFS than mFFX (mean difference: 2.8 months, *p* < 0.001). Furthermore, S-1 was ranked best for lowest toxicities in overall and haematological grade 3/4 [5]. Moreover, Cytochrome P450 2A6, as the key enzyme in converting tegafur to 5-FU, is more active in Asian popuaption than that of western ethnic groups [8, 9] . Therefore, S-1 maybe the most suitable adjuvant regimen for resection patients in an Asian population [10]. However, the optimal duration of S1 administration for resectable PDAC is still unknown. Recent evidence suggests that S-1 for 8-courses should remain as standard adjuvant chemotherapy for stage II gastric cancer [11] and was feasible and may be a promising treatment for those with resected biliary tract cancer (BTC) [8]. Moreover, the most common grade 3–4 adverse event was neutropenia, observed in 46 (16%) patients in the eight-course group and 51 (17%) patients in the four-course group [11]. Besides the good toleration of S-1, the pharmacokinetics of orally administered S-1 were similar to those of continuously infused fluorouracil, a time-dependent drug. Therefore, the 28-day continuous dosing of S-1 might be theoretically advantageous from the viewpoint of exposure time to an anti-tumour agent by the cancer cells [7, 12]. Considering this, we hypothesized that the PDAC patients who had undergone surgery may profit from the chemotherapy regimens, including a longer duration of S-1 administration, so as that of the gastric cancer or BTC. Hence this study aimed to investigate the potential benefit of more than 4 courses of S1 adjuvant chemotherapy for patients with PDAC after surgery.

## Methods

### Cohort development

We referred our previous research [13] to develop cohorts. We enrolled consecutive patients who underwent S-1 adjuvant chemotherapy following curative pancreatectomy between January 2016 and December 2018 at the Department of Hepatobiliary Pancreatic Surgery in Changhai Hospital (Shanghai, China). With respect to the inclusion criteria: (1) patients who underwent surgery with curative intent and S-1 adjuvant chemotherapy; (2) patients who were able to start chemotherapy within 8 weeks after surgery; (2) age ≥ 20 years and < 80 years; (3) adequate oral intake; (4) adequate bone marrow function, adequate liver function and adequate renal function for adjuvant chemotherapy. The exclusion criteria for this study were as follows: (1) patients with intraoperative metastasis or macroscopic evidence of margin involvement (R2); (2) patients who received neoadjuvant chemotherapy or radiotherapy; (3) patients with other malignancies in the past; and (4) patients who died within 90 days; (5) patients’ S-1 could not been administrated within 8 weeks after the surgery; (6) patients with incomplete follow-up data, (7) patients who did not complete 4 courses of S-1 administration. The therapy schedule was approved by the Institutional Review Board of Changhai Hospital and Hospital and all informed consent was obtained from participants or from the legally authorized representatives for participating in this study for chemotherapy for the start of oncological treatment, and the study was conducted in accordance with the Declaration of Helsinki and national guidelines.

### Treatment

S-1 (tegafur, gimeracil, oteracil potassium; Taiho Pharmaceutical, Tokyo, Japan) was administered within 8 weeks after the surgery. An oral dose of 80 mg/m^2^ S-1 was given every day on days 1 to 28 of a 6-week course. The total dose was based on the patient’s body surface area as follows: < 1.25 m2, 80 mg; 1.25–1.5 m2, 100 mg; > 1.5 m2, 120 mg. The total dose was calculated for each course. The course was repeated for at least 6 months (4 courses) until unacceptable toxicity, or refusal by the patient to undergo further treatment. Those patients who had completed 4 courses may continue 1 to 4 courses therapy based on the physicians’ recommendation and informed consent of the patients. The physicians recommended those patients who had high risks of tumor recurrence, such as R1, late stage, high level of preoperative serum carbohydrate antigen 19–9 (CA19–9), prolonged the duration of S-1 therapy. Toxicity was categorized and recorded according to the common toxicity criteria of the National Cancer Institute (version 4.0). The study categories the patients as 4 courses cohort and > 4 courses cohort. Relative dose intensity (RDI) was defined as the proportion of actual dose intensity received to the planned dose intensity.

### Follow-up protocol and analyzed variables

We refereed the follow-up protocol and analyzed variables reported in our previous research [13]. The main outcome was over survival (OS). Secondary outcomes included adverse effects (AEs) and relapse-free survival (RFS). The institutional follow-up was jointly completed by department follow-up specialists, and the third-party professional data were provided by LinkDoc Technology Co. Ltd. (Beijing, China). Postoperative follow-up CT/MRI scanning were performed at 3, 6, 12 months for the first year and every 6 months following that. Serum CA19–9 were conducted every 3 months for 5 years. The methods for follow-ups included outpatients visits, contacting by phone, mail, chatting software or address. The general information of follow-ups included adjuvant therapy, recurrence, the cause of death, et al. OS was defined as the time from operation to death. RFS was defined as the time from operation to first site recurrence, including regional recurrence and systematic recurrence. Patients who were still alive at the cut-off date of follow-ups were censored at the date at which they were last confirmed to be alive. We defined loss to follow up as no-show on the clinical follow-ups or the patients or their family members cannot be contacted by phone, mail or address. For all patients, the following demographic and clinic pathological variables derived from our perspective data center, were recorded: sex, age, tumor location (head/neck/uncinate, body/tail), preoperative serum CA19.9, perineural invasion, lymphovascular invasion, R status (R1 or R0), tumor grade (G1/2 or G3/4), and information on postoperative adjuvant therapy and survival time. Furthermore, TNM staging was recorded according to the 8th edition of AJCC Cancer Staging Manual for Pancreatic Cancer [14].

### Statistical analysis

The statistics analysis was also refered to our previous research [13]. Categorical data are presented as percentages and were examined using the chi-squared test or Wilcoxon rank-sum test Univariate and multivariable Cox regression analyses were performed to identify independent prognostic factors, and hazard ratios (HRs) were calculated. Variables with *P* value < 0.05 in univariate analyses were included in multivariate analyses using a forward selection algorithm. OS curves and RFS curves were assessed using the Kaplan–Meier method and log-rank test. For all analyses, a two-tailed *P* < 0.05 was considered statistically significant. Analyses were performed using SPSS version 25.0 (IBM Corp., Armonk, NY, USA).

## Results

### Study population

**Fig. 1:**
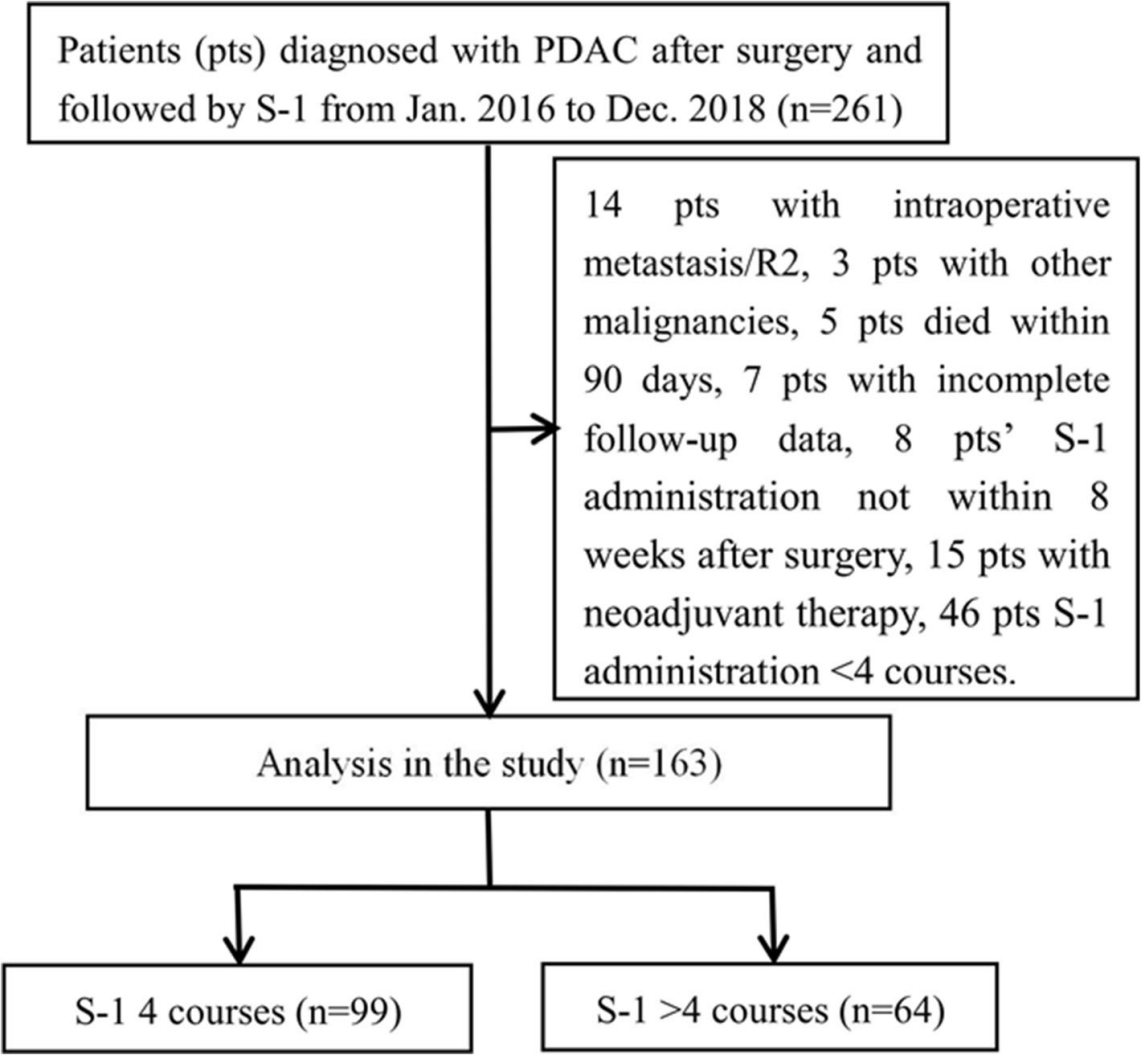
The flowchart of patients selection in the study

Of the 261 consecutive patients in our study, 98 were excluded because they had intraoperative metastasis or R2 (*n* = 14), received neoadjuvant chemotherapy or radiotherapy (*n* = 15), had other malignancies in the past (*n* = 3), died within 90 days (*n* = 5), could not been administrated within 8 weeks after the surgery (*n* = 8), were lost to follow-up (*n* = 7) or did not completed 4 courses of S-1 administration. All patients enrolled were of yellow race. The 4 courses cohort comprised 99 patients, whereas > 4 courses cohort consisted of 64 patients (Fig. 1). All of 163 patients, 99 (60.7%) patients took S-1 4 courses, 8 (4.9%) patients took 5 courses, 11 (6.7%) patients as 6 courses, 22 (13.5%) patients as 7 courses and 23 (14.1%) patients as 8 courses. Table 1 shows no significant differences in tumor characteristics were found between the two cohorts.

**Table 1:** Association between clinicopathological features and S-1 administration duration

(%)	S-1 administration duration		***P***
	4 courses	>4 courses	
**Total**	99 (60.7)	64 (39.3)	
**Sex**			0.473
Male	50 (50.5)	36 (56.3)	
Female	49 (49.5)	28 (43.8)	
**Age (years)**			0.692
≤ 65	51 (51.5)	35 (54.7)	
> 65	48 (48.5)	29 (45.3)	
**CA19.9(IU/mL)**			0.252
< 37	28 (28.3)	13 (20.3)	
≥ 37	71 (71.7)	51 (79.7)	
**Tumor location**			0.295
Head/neck/uncinate	65 (65.7)	47 (73.4)	
Body/tail	34 (34.3)	17 (26.6)	
**Grade**			0.865
1/2	86 (86.9)	55 (85.9)	
3	13 (13.1)	9 (14.1)	
**Lymphovascular invasion**			0.772
Without	77 (77.8)	51 (79.7)	
With	22 (22.2)	13 (20.3)	
**Perineural invasion**			0.865
Without	13 (13.1)	9 (14.1)	
With	86 (86.9)	55 (85.9)	
**T stage**			0.814
1	18 (18.2)	12 (18.8)	
2	55 (55.6)	38 (59.4)	
3	26 (26.3)	14 (21.9)	
**N stage**			0.473
0	59 (59.6)	32 (50.0)	
1	33 (33.3)	27 (42.2)	
2	7 (7.1)	5 (7.8)	
**TNM stage**			0.794
I	44 (44.4)	25 (39.1)	
II	48 (48.5)	34 (53.1)	
III	7 (7.1)	5 (7.8)	
**R status**			0.072
0	90 (90.9)	52 (81.3)	
1	9 (9.1)	12 (18.8)	
Chemotherapy regimens			0.528
S1 only	87 (87.9)	53 (82.8)	
S1 + gemcitabine	6 (6.1)	4 (6.3)	
S1 + other drugs	6 (6.1)	7 (10.9)	

*Abbreviation*: *TNM* Tumor–node–metastasis

### Prognostic analysis

**Fig. 2:**
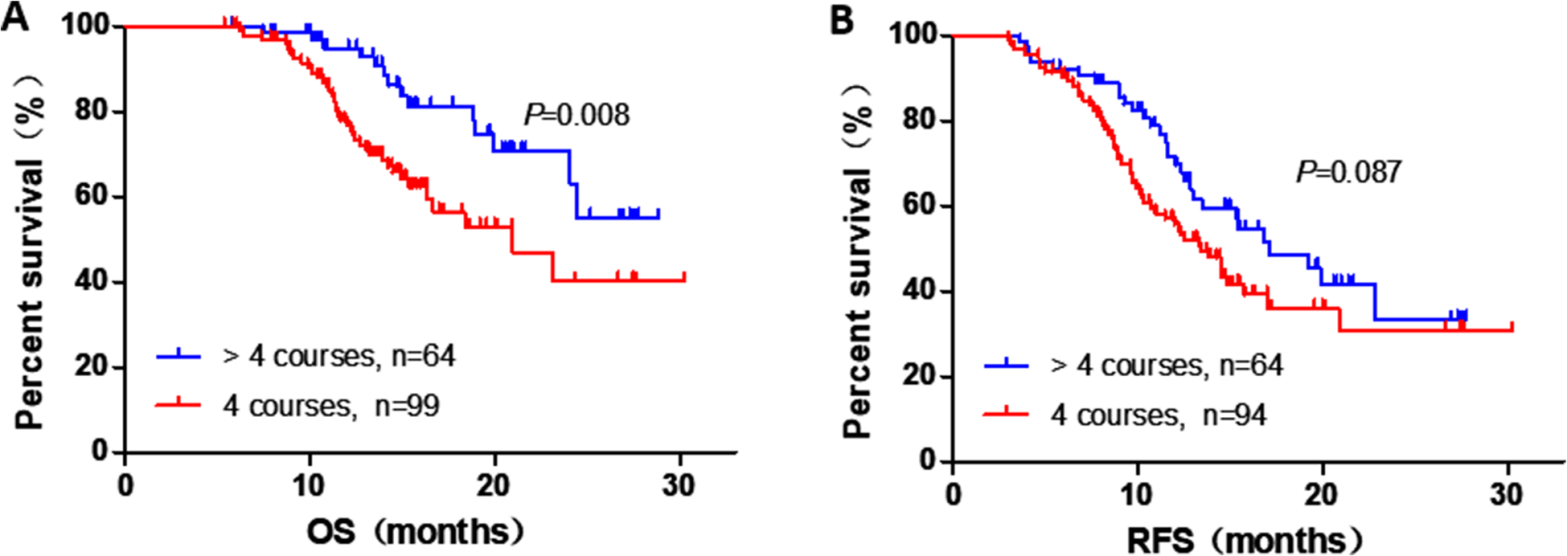
Kaplan-meier diagrams showing OS for S-1 administration duration of > 4 courses and 4 courses, excluding patients who had died within 3 months (**A**), RFS for S-1 administration duration of > 4 courses and 4 courses, excluding patients who had tumor recurrence within 3 months (**B**). *P*-values for log rank test are shown in each panel

The median OS after surgery was 24.4 months in this study. We performed Cox regression analysis to examine the effect of postoperative clinicopathological parameters and duration of S-1 administration on prognosis. Univariate analyses revealed that TNM stage (stage II vs. I: hazard risk [HR], 1.913; 95% confidence interval [CI], 1.014–3.609; *P* = 0.045), duration of S-1 administration (4 vs. **>** 4 courses: HR, 2.248; 95% CI, 1.178–4.291; *P* = 0.014) and tumor grade (G3 vs. G1/2: HR, 3.419; 95% CI, 1.713–6.823; *P* < 0.001) were significantly with OS. Furthermore, multivariate analysis confirmed that TNM stage (stage II vs. I: HR, 2.125; 95% CI, 1.164–4.213; *P* = 0.015), duration of S-1 administration (4 vs. > 4 courses: HR, 3.113; 95% CI, 1.531–6.327; *P* = 0.002) and tumor grade (G3 vs. G1/2: HR, 3.887; 95% CI, 1.922–7.861; *P* < 0.001) were also independent prognostic factors (Table 2). And the univariate and multivariable analyzes of all variables evaluated were showed in Supplementary Table 1. Figure 2A showed the OS curves which excluded the who had died within 3 months, and the median OS for S-1 administration duration 4 courses was 20.9 months, whereas that for **>** 4 courses did not reached (*P* < 0.001). Furthermore, RFS of the 2 cohorts was also been compared, and Fig. 2B showed RFS excluded patients with tumor recurrence within 3 months (4 vs. **>** 4 courses, *P* = 0.087).

**Table 2:** Univariate and multivariate Cox regression analyses of clinicopathological features associated with OS of patients with PDAC

	Univariate		Multivariate	
	HR (95% CI)	*P*	HR (95% CI)	*P*
**TNM stage** II vs. I	1.913 (1.014–3.609)	0.045	2.215 (1.164–4.213)	0.015
**S1 (**courses) 4 vs. > 4	2.248 (1.178–4.291)	0.014	3.113 (1.531–6.327)	0.002
**Grade** G3 vs. G1/2	3.419 (1.713–6.823)	< 0.001	3.887 (1.922–7.861)	< 0.001

*Abbreviations*: *CI* Confidence interval, *HR* Hazard ratio, *TNM* Tumor–node–metastasis

**Fig. 3:**
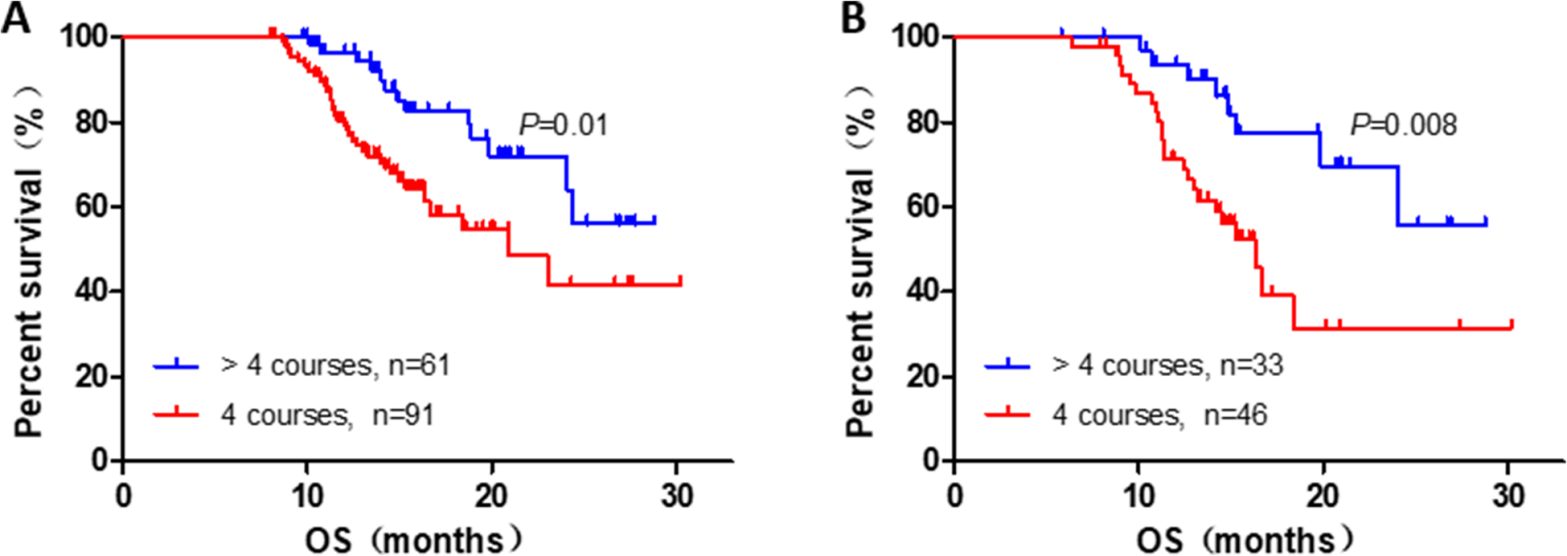
Kaplan-meier diagrams showing OS of total (**A**), stageII (**B**) for S-1 administration duration of > 4 courses and 4 courses, excluding patients who had died within 8 months. *P*-values for log rank test are shown in each panel

In further, to avoid the potential bias related with S-1 intake duration, we investigated the prognostic implication of S-1 intake duration in the patients who can survive beyond 8 months. We found that the OS of patients in **>** 4 courses cohort was significantly prolonged compared with that of 4 courses cohort (4 vs. **>** 4 courses: HR, 2.284; 95% CI, 1.197–4.358; *P* = 0.012, Fig. 3A). In the subgroup analysis, we found patients in TNM stage (4 vs. **>** 4 courses: HR, 2.906; 95% CI, 1.275–6.623; *P* = 0.011, Fig. 3B), T3 (4 vs. **>** 4 courses: HR, 5.277; 95% CI, 1.110–25.1; *P* = 0.037), N0(4 vs. **>** 4 courses: HR, 3.117; 95% CI, 1.038–9.357; *P* = 0.043), N1 (4 vs. **>** 4 courses: HR, 2.688; 95% CI, 1.067–6.673; *P* = 0.036) could acquire more benefit on prognosis under the S-1 administration duration of **>** 4 courses compared with that of 4 courses.

### Treatment adherence and adverse events

The median RDI of administered S-1 were 83.3 and 85.9% in the 4 courses cohort and > 4 courses cohort, respectively, which were significantly different (*P* < 0.001). Table 3 summarizes the adverse events (AEs) in each treatment cohort. The incidence of AEs of grade1–2 was 37.4% in 4 courses cohort and 39.1% in the > 4 courses cohort (*P* > 0.05). The incidence of grade 3 AEs was 15.2% in 4 courses cohort and 15.6% in the > 4 courses cohort, showing no significant difference (*P* > 0.05). No grade 4 or 5 AEs were observed in both cohort.

**Table 3:** dverse events in each cohort

Adverse events (%)	4 courses (*n* = 99)		> 4 courses (*n* = 64)	
	Grade1–2	Grade3	Grade1–2	Grade3
Overall	37 (37.4)	15 (15.2)	25 (39.1)	10 (15.6)
Leukopenia	18 (18.2)	2 (2.0)	10 (15.6)	2 (3.1)
Neutropenia	23 (23.2)	3 (3.0)	11 (17.2)	2 (3.1)
Thrombocytopenia	5 (5.1)	1 (1.0)	3 (4.7)	2 (3.1)
Anemia	14 (14.1)	0 (0)	9 (14.1)	1 (1.6)
Elevated AST level	9 (9.1)	2 (2.0)	6 (9.4)	2 (3.1)
Elevated ALT level	7 (7.1)	1 (1.0)	4 (6.3)	0 (0)
Elevated total bilirubin level	13 (13.1)	1 (1.0)	6 (9.4)	1 (1.6)
Oral mucositis	7 (7.1)	1 (1.0)	3 (4.7)	1 (1.6)
Nausea/Vomiting	14 (14.1)	2 (2.0)	7 (10.9)	2 (3.1)
Fatigue	21 (21.2)	1 (1.0)	12 (18.8)	1 (1.6)
Diarrhea	12 (12.1)	1 (1.0)	8 (12.5)	2 (3.1)

*AST* Aspartate aminotransferase, *ALT* Alanine aminotransferase

## Discussion

Recently, a network meta-analysis showed that S-1 or mFFX were the best adjuvant therapy regimen for prolonging the OS after pancreatectomy [5]. Another network meta-analysis indicated that S-1, as a regimen of adjuvant chemotherapy, ranked the best in terms of prolonging OS of 1- and 3-year with the least toxic [15]. In addition, compared with other chemotherapy, patients are more tolerated and adherent to S-1 [16]. Besides, Asian patients are with ethinic strength for converting tegafur to 5-FU [16]. Thus, the physician are more inclined to prescribe S-1 for the patients in real world practice to improve the therapeutic effect. JASPAC 01 trial demonstrated superior OS and RFS in patients who received S-1 for duration of 4 courses [8]. However, it is not clear whether it is necessary to extend the duration of adjuvant chemotherapy prolonged to improve the dismal 5-year survival rate of PDAC. Meanwhile, studies suggested that S-1 administration of 8 courses was a beneficial treatment for patients with gastric cancer and BTC after surgery [8, 11]. Our studies proved that the OS was significantly prolonged for patients who had S-1 administration for more than 4 courses compared with 4 courses. In the extended analysis, the survival benefit in subgroup patients of T3, N0/1, stage II were significant, indicating that patients in early stage or relatively late stage could not obtain sufficient benefit from the prolonged S-1 administration. This conclusion also consistent with the results of a phase 3, open-label, randomized controlled, non-inferiority trial in patients with gastric cancer stage II [11]. However, there was only a trend of prolonged RFS survival for S-1 intake beyond 4 courses, which was same as gastric cancer in previous study [11]. Therefore, this study confirmed thatS-1 advantages of continuous anti-tumor dosing against the cancer cells for good prognosis. At the same time, prolonged duration of therapy were not increased the occurrence of adverse effects, which has been proved by other researches [8, 11].

Although the conclusion was derived from a retrospective research, we had endeavored to reduce bias to solid the results by excluding patients who died within 8 months after surgery [14]. All the data analyzed were derived from the perspective data center, which was managed by experienced engineers. For example, the RDI, highly correlated with therapeutic effect [17], of the two cohort are relatively higher compared with that of the primary studies [8, 16], which also indicated the good quality of patients management in our study.

Above all, it indicated that extended duration of S-1 intake may change the gloomy survival situation of patients with PDAC, especially for Asian population in stage II.

The current study has several limitations. First, our study has the intrinsic shortcomings of any retrospective study. Although the baseline clinicopathological features of the two cohorts were relatively balanced, there is an inherent bias in the selection of patients for extended therapy, which based on different physicians’ recommendation and informed consent of the patients. Considering the small number of this study, we did not conduct propensity score matching to eliminate baseline bias. Second, due to the retrospective nature of the study. Compared with 4 courses cohort, the > 4 courses cohort including 19 patients who had S-1 for 5 to 6 courses may weaken the statistics significance of this study. Third, that results of S1 therapy are generally related to Asian population. Therefore, prospective study needs to be carried out to validate the conclusion. Last, our current conclusion were only based on data from a single center and multiple center analysis is ongoing.

## Conclusion

Our findings indicate that more than 4 courses of adjuvant S-1 therapy for resected PDAC was feasible. Prolonged duration of S-1 intake is beneficial to prognosis of patients with PDAC resection. The exact optimal courses of adjuvant S-1 treatment is unclear and requires further studies.

## Supplementary Information

**Additional file 1: Supplementary Table 1.** Univariate and multivariable Cox regression analyses of all clinicopathological features evaluated of patients with PDAC.

## Data Availability

The datasets used and/or analyzed during the current study are available from the corresponding author on reasonable request.
