# Supplementary material for: Comparison of 4- and 4 plus-courses S-1 administration as adjuvant chemotherapy for pancreatic ductal adenocarcinoma
Source: BMC Cancer. 2021 May 26;21:612. doi: 10.1186/s12885-021-08380-9 (PMC8152347; doi:10.1186/s12885-021-08380-9)
Supplement: Supplementary file 1 — Additional file 1: Supplementary Table 1. Univariate and multivariable Cox regression analyses of all clinicopathological features evaluated of patients with PDAC. [file 12885_2021_8380_MOESM1_ESM.docx]

**Supplementary table 1** Univariate and multivariable Cox regression analyses of all clinicopathological features evaluated of patients with PDAC

|  | **Univariate** | | **Multivariate** | |
| --- | --- | --- | --- | --- |
|  | HR (95% CI) | P | HR (95% CI) | P |
| **TNM stage**Ⅱ/Ⅲvs.Ⅰ | 1.913(1.014–3.609) | 0.045 | 2.215 (1.164–4.213) | 0.015 |
| **S1 (**courses)  4 vs. >4 | 2.248(1.178–4.291) | 0.014 | 3.113 (1.531–6.327) | 0.002 |
| **Grade**  G3 vs. G1/2 | 3.419(1.713–6.823) | <0.001 | 3.887 (1.922–7.861) | <0.001 |
| **Perineural invasion** |  |  |  |  |
| With v.s. without | 1.405(0.556-3.549) | 0.472 |  |  |
| **Lymphovascular invasion** |  |  |  |  |
| With v.s. without | 1.170(0.622-2.202) | 0.626 |  |  |
| **R status** |  |  |  |  |
| R1 v.s. R0 | 0.816(0.324-2.056) | 0.667 |  |  |
| **Chemotherapy regimens** |  |  |  |  |
| S1+gemcitabine v.s. S1 only | 0.743(0.166–3.328) | 0.698 |  |  |
| S1+other drugs v.s. S1 only | 0.908(0.326–2.530) | 0.854 |  |  |
| **CA19.9（IU/mL）** |  |  |  |  |
| ≥37 v.s. <37 | 1.420(0.709–2.845) | 0.323 |  |  |

Abbreviations: CI, confidence interval; HR, hazard ratio; TNM, tumor–node–metastasis
